# α2,3-Linked Sialic Acids Are the Potential Attachment Receptor for Shaan Virus Infection in MARC-145 Cells

**DOI:** 10.1128/spectrum.01256-22

**Published:** 2022-08-04

**Authors:** Seong Sik Jang, Ji Yeong Noh, Min Chan Kim, Hyun A. Lim, Min Suk Song, Hye Kwon Kim

**Affiliations:** a Department of Biological Sciences and Biotechnology, College of Natural Science, Chungbuk National University, Cheongju, Republic of Korea; b Department of Microbiology, College of Medicine and Medical Research Institute, Chungbuk National University, Cheongju, Republic of Korea; Thomas Jefferson University

**Keywords:** Shaan virus, bat paramyxovirus, glycan microarray, magnetic bead, neuraminidase, receptor, sialic acid

## Abstract

Shaan virus (ShaV), a novel species of the genus *Jeilongvirus*, family *Paramyxoviridae*, was isolated from an insectivore bat (Miniopterus schreibersii) in Korea in 2016. ShaV particles contain a hemagglutinin-neuraminidase (HN) glycoprotein in their envelope that allows the virus to target cells. Typically, diverse paramyxoviruses with HN glycoprotein are reported to interact predominantly with sialic acids, but there are no studies of receptors for ShaV. In this study, the identification of potential receptors for ShaV was demonstrated using sialidase treatments, glycan microarray, magnetic bead-based virus binding assay, and neuraminidase inhibitor treatments. Pretreatment of MARC-145 cells with sialidase, which cleaves α2,3-linked sialic acids, showed higher inhibition of viral infection than α2,6-linked-specific sialidase. These data were supported by the binding of ShaV to predominantly α2,3-linked sialylated glycans in the screening of sialyl linkage patterns through glycan microarray. To further confirm the direct interaction between ShaV and α2,3-linked sialic acids, ShaV was incubated with α2,3- or α2,6-linked sialylated glycans conjugated to magnetic beads, and binding signals were detected only for α2,3-linked sialylated glycans. In addition, the potential of sialic acids as a receptor was demonstrated by the viral replication inhibitory effect of the neuraminidase inhibitor 2,3-dehydro-2-deoxy-*N*-acetylneuraminicacid (DANA) in the mature virion release steps. Collectively, these results support that α2,3-linked sialic acids are the potential receptor for ShaV infection in MARC-145 cells.

**IMPORTANCE** Bats host major mammalian paramyxoviruses, and novel paramyxoviruses are increasingly being reported around the world. Shaan virus (ShaV), from the genus *Jeilongvirus*, family *Paramyxoviridae*, has a potential attachment glycoprotein, HN. Here, we identify that ShaV binds to sialic acid and demonstrate that α2,3-linked sialic acids are the potential receptor for ShaV infection. The presented data regarding ShaV receptor specificity will enable studies into the viral tropism for the host and contribute to the development of new antiviral strategies targeting viral receptors.

## INTRODUCTION

Bats are considered potential natural host animals for major mammalian paramyxoviruses of the family *Paramyxoviridae* (1). Active virus surveillance is ongoing worldwide. In fact, over the past several decades, diverse paramyxoviruses found in bats have been identified to be closely related to human and livestock pathogens, such as measles, mumps, parainfluenza, Newcastle disease, henipavirus, and respiratory syncytial virus (2). In South Korea, bat virus screening was performed with fecal samples collected from natural bat habitats, and Shaan virus (ShaV) of a novel paramyxovirus from insectivorous bats (Miniopterus schreibersii) was identified (3).

ShaV was isolated from the MARC-145 cell line, which is a clone of the MA-104 cell line derived from African green monkey kidney epithelial cells (4). ShaV, which belongs to the genus *Jeilongvirus*, subfamily *Orthoparamyxovirinae*, and family *Paramyxoviridae*, has a potential attachment glycoprotein, hemagglutinin-neuraminidase (HN), embedded in its envelope (5). Paramyxovirus HN glycoprotein is present in Sendai virus, mumps virus, parainfluenza virus 5 (PIV5), human parainfluenza viruses 1 to 4 (HPIV1 to HPIV4), and Newcastle disease virus (NDV). The HN glycoprotein plays a key role in viral infection. In fact, this glycoprotein binds to sialic acid-containing receptors on cell surfaces for virus attachment to cells. In addition, the activity of neuraminidase removes sialic acid from progeny virus particles, which enables the prevention of virus self-aggregation and efficient virus spread (6, 7).

HN consists of a domain that includes an N-terminal cytoplasmic tail, a single N-terminal transmembrane (TM) domain, and a C-terminal large ectodomain. The ectodomain consists of a helical stalk and a globular head that performs both receptor binding and cleavage activity. According to a model based on structural studies, the HN glycoproteins of PIV5 and NDV convert the active form of the tetramer after interaction with sialic acid as a receptor (8–11). This conversion is thought to activate the viral fusion protein and promote fusion between the virus and the cell membrane, leading to delivery of the nucleocapsid into the cytoplasm (7, 12).

The most common mammalian sialic acid residues (Neu5Ac or Neu5Gc) are present on cell surface gangliosides and *N*- and *O*-glycoproteins, which are linked to subterminal galactose by α2,3 or α2,6 linkages (13). Many studies have reported the interaction between HN glycoprotein and sialic acids in paramyxoviruses. For example, studies with enzymatic treatment of cells revealed that NDV binds to both a2,3- and a2,6-linked sialic acids (14). Additionally, a solid-phase binding assay revealed that HPIV1 binds preferentially to α2,3-linked sialic acids, whereas HPIV3 binds strongly to α2,3- and α2,6-linked sialic acids (15).

Recently, the detection of novel paramyxoviruses closely related to the genus *Jeilongvirus* and ShaV has been reported not only in bats but also in a broad host range (16–19). This may indicate that ShaV should be further studied to prepare the potential for interspecies transmission. In this context, the identification of host receptors plays an important role in understanding viral host range, tissue affinity, and viral pathogenesis (20, 21). However, the role of sialic acids as a receptor for ShaV remains unclear. Therefore, we sought to determine whether ShaV HN is a sialic acid binding protein and identify the type of sialyl linkage present in the host cell for ShaV binding.

## RESULTS

### Reduction of ShaV infection by sialidase treatment.

To determine the effect of sialidase treatment, the antigenome and total ShaV RNAs were quantified. First, to establish a harvest time point for sialidase-treated cells, MARC-145 cells were inoculated with ShaV for different incubation times using different multiplicities of infection (MOIs). The amount of viral antigenome plateaued from 18 h postinoculation (hpi) at MOIs of 0.01, 0.1, and 1. Further, the amount of total viral RNAs increased from 24 hpi at MOIs of 0.01, 0.1, and 1 (see Fig. S1 in the supplemental material). Therefore, 12 hpi was identified as the time point of sialidase treatment for ShaV antigenome and total RNAs detection to evaluate the efficiency of virus-host interactions by avoiding plateau conditions in gene expression levels.

**FIG 1:**
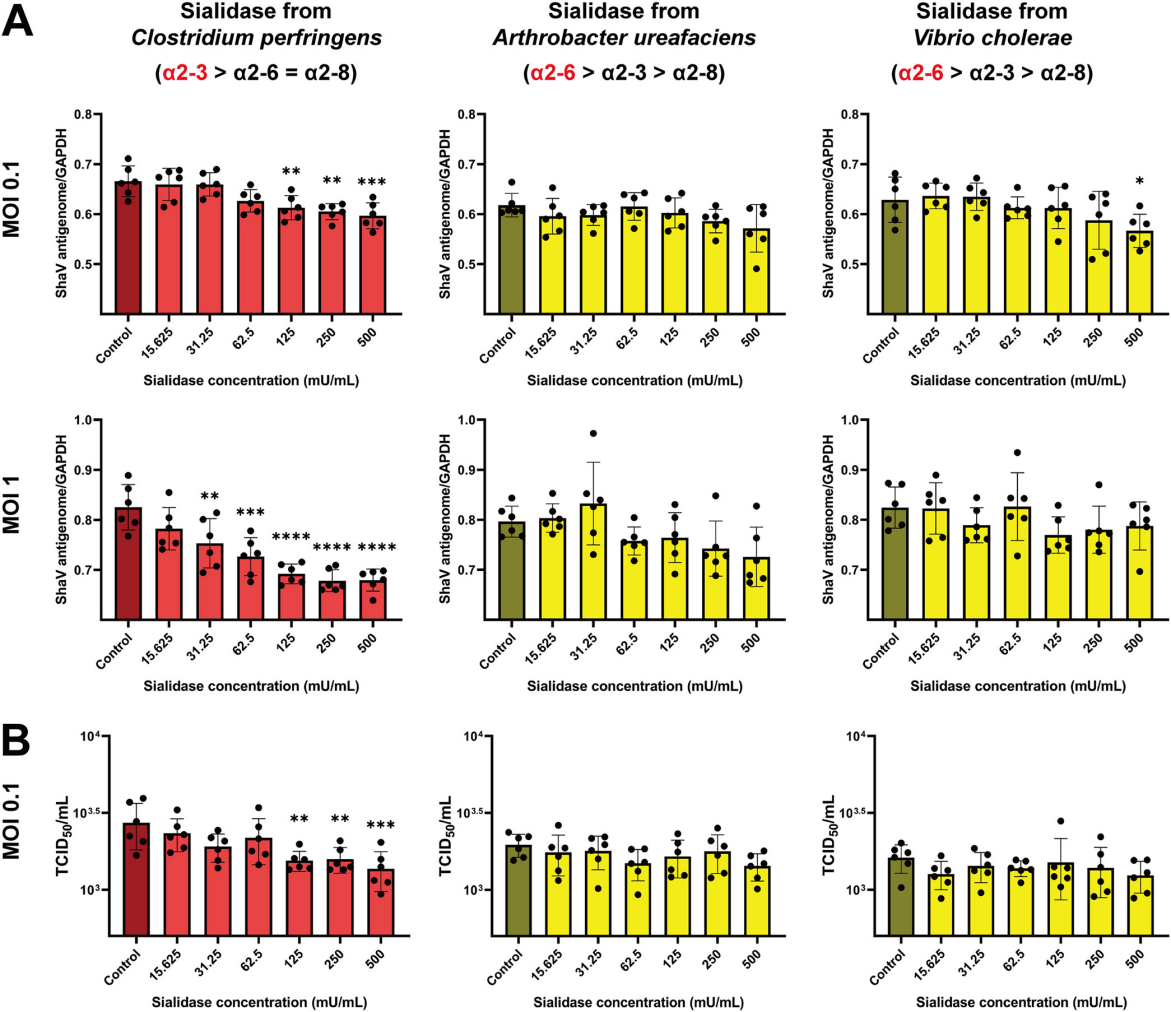
Quantitative analysis of ShaV antigenome and total RNAs by sialidase treatment. (A) Sialidase-treated MARC-145 cells with 0.1 MOI and 1 MOI of ShaV. The relative expression of viral antigenome normalized with monkey GAPDH gene quantified by real-time qPCR. (B) Sialidase-treated MARC-145 cells infected with 0.1 MOI of ShaV. The relative expression of total viral RNAs quantified by RT-qPCR and *C_q_* values converted to TCID_50_/mL via standard curve. Control cells were infected with ShaV only. Sialidase from Clostridium perfringens preferentially cleaves α2,3 linkage (red) at higher rate than other linkages, while sialidases from Arthrobacter ureafaciens and Vibrio cholerae preferentially cleave α2,6 linkages (yellow). The experiments were repeated six times. Error bars represent the corresponding SD. The data were analyzed by one-way analysis of variance (ANOVA) with Dunnett’s multiple-comparison test. *, *P* < 0.05; **, *P* < 0.01; ***, *P* < 0.001; ****, *P* < 0.0001.

Thereafter, to assess the role of sialic acid as a ShaV receptor, MARC-145 cells were treated with sialidase from Arthrobacter ureafaciens, Vibrio cholerae, and Clostridium perfringens prior to infection. All enzymes can cleave sialic acid residues of α2,3, α2,6, or α2,8 linkage. Sialidase from Clostridium perfringens preferentially cleaves the α2,3 linkage at a higher rate than other linkages (α2,3 > α2,6 = α2,8), while sialidases from Arthrobacter ureafaciens and Vibrio cholerae preferentially cleave the α2,6 linkages (α2,6 > α2,3 > α2,8). To determine the effect of sialidase on ShaV infection, antigenome quantification was performed at MOIs of 0.1 and 1. Treatment with sialidase from Clostridium perfringens reduced ShaV infection in a concentration-dependent manner from 125 to 500 mU/mL at an MOI of 0.1 and from 31.25 to 500 mU/mL at an MOI of 1. Although sialidase from Vibrio cholerae only reduced viral infection at 500 mU/mL (MOI of 0.1), sialidase from Arthrobacter ureafaciens did not reduce viral infection (Fig. 1A).

To further determine the effect of sialidase, we also detected total ShaV RNAs in the same way as in the above-described experiment. A standard curve was used to estimate the virus concentration through conversion from the quantification cycle (*C_q_*) value of total viral RNAs to 50% tissue culture infective dose (TCID_50_) per milliliter (Fig. S2). As shown in Fig. 1B, treatment with sialidase from Clostridium perfringens reduced ShaV infection in a concentration-dependent manner from 125 to 500 mU/mL at an MOI of 0.1. However, treatment with sialidase from Arthrobacter ureafaciens and Vibrio cholerae did not reduce ShaV infection compared with the untreated control.

### Identification of sialidase effects by plaque reduction assay.

**FIG 2:**
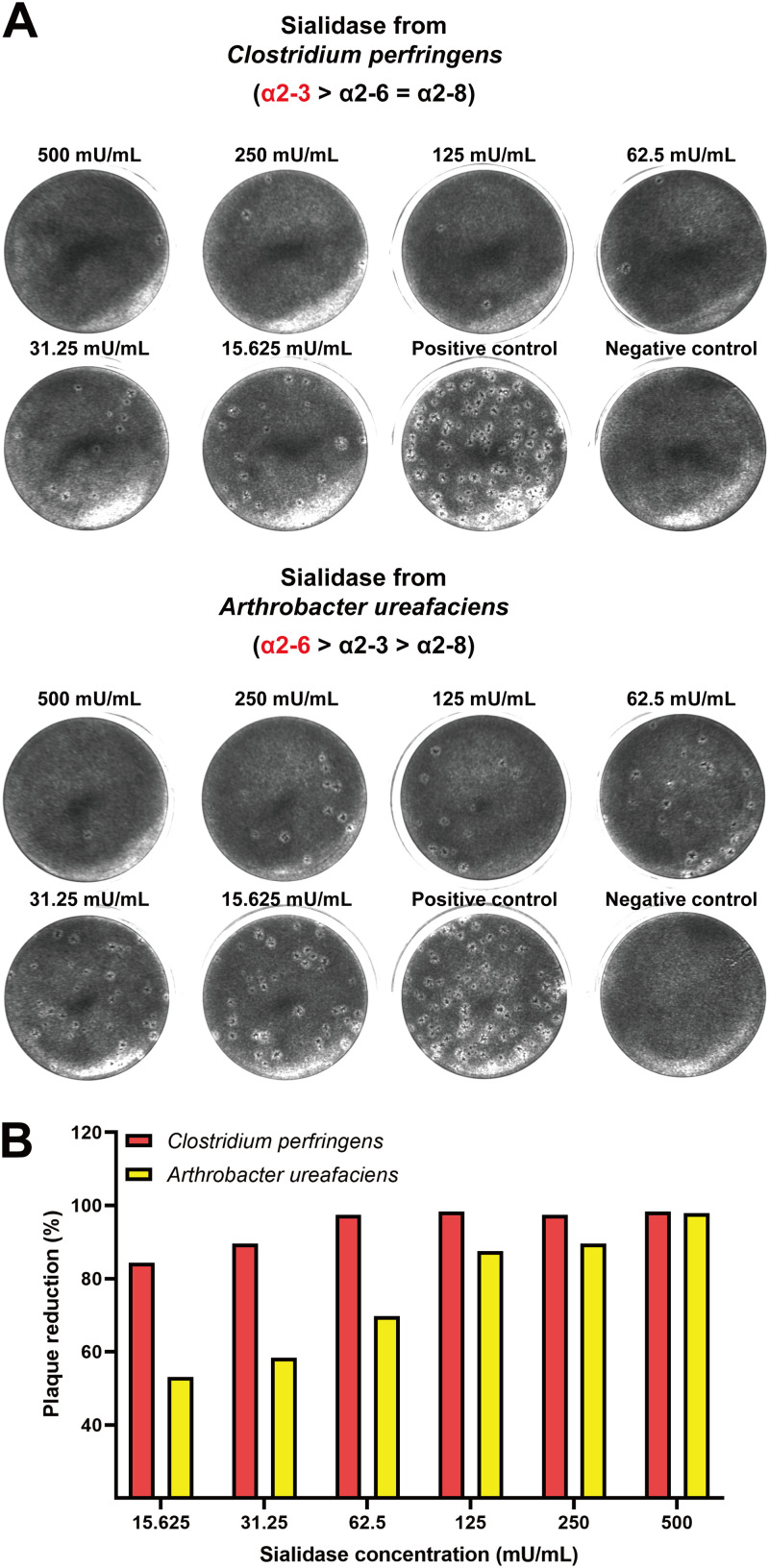
(A) Reduction of virus infectivity by sialidase from Clostridium perfringens and Arthrobacter ureafaciens was determined by plaque assay. MARC-145 cells were inoculated with lysates from cells infected with ShaV at an MOI of 0.1 after sialidase treatment. One hour afterward, the viral inoculum was removed, and cells were overlaid with agarose. After 7 days, the cells were stained, and images were captured. (B) The plaque numbers at different concentrations were counted and compared to controls to determine the reduction rate of plaque formation.

Reduction of virus infectivity by two sialidases from Arthrobacter ureafaciens and Clostridium perfringens was further determined by plaque assay. As shown in Fig. 2A, ShaV plaque formation decreased in a concentration-dependent manner for both sialidases. However, treatment with sialidase from Clostridium perfringens significantly reduced plaque formation compared to sialidase from Arthrobacter ureafaciens. Sialidase from Clostridium perfringens reduced the number of plaques by 84.3 to 98.3%, whereas sialidase from Arthrobacter ureafaciens reduced the number of plaques by 53.1 to 97.9% (Fig. 2B).

### Screening of ShaV binding patterns using glycan microarray.

**FIG 3:**
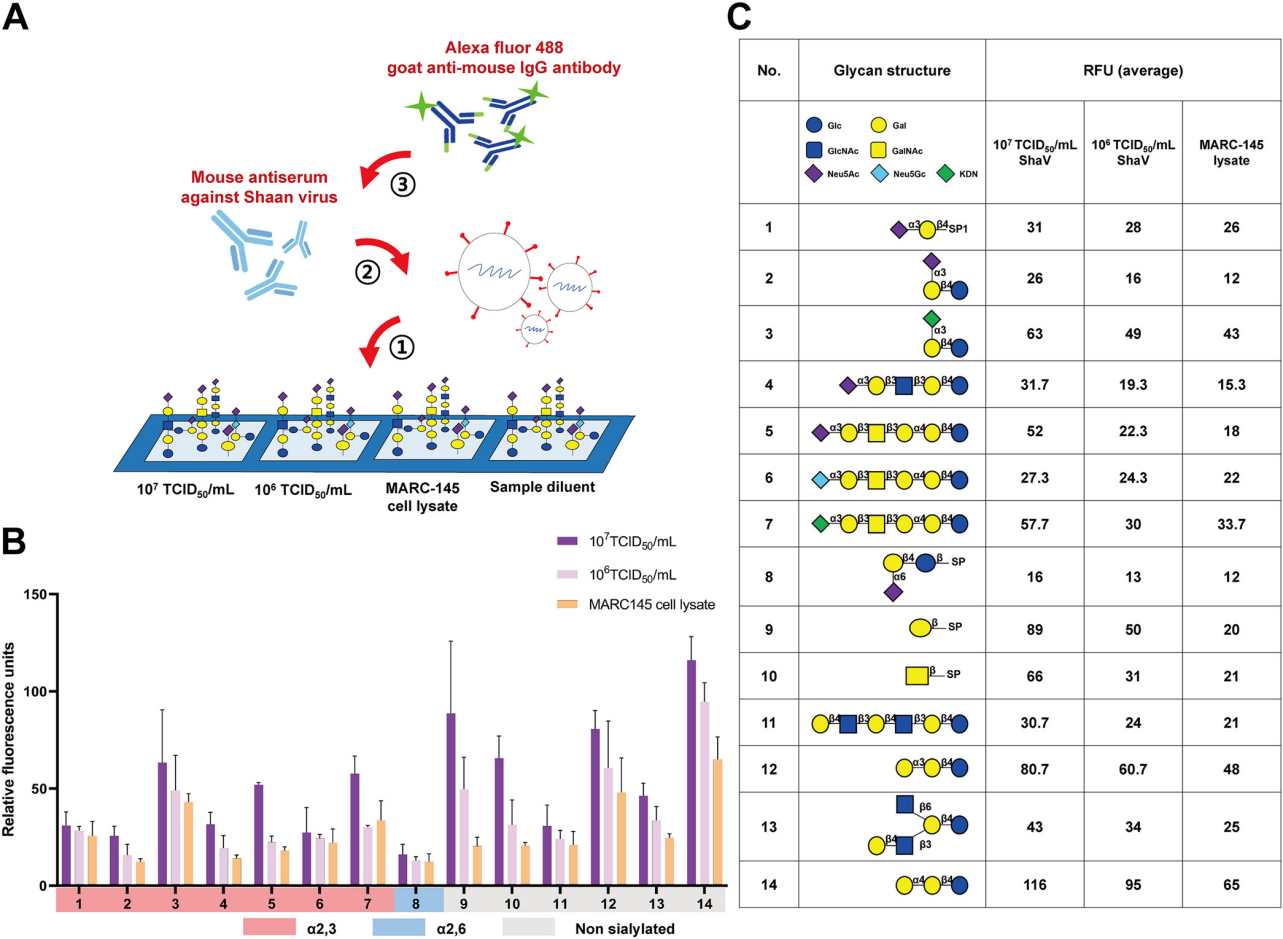
(A) Schematic illustration of sandwich-based glycan microarray. ShaV of different titers, MARC-145 cell lysate, and sample diluent used as control were added to slide printed with glycans and incubation. After incubation, mouse antiserum against ShaV was added as primary antibody. Then, anti-mouse antibody conjugated with Alexa fluor 488 was added, and the slide was read using a microarray scanner. (B) Glycan microarray analysis of the binding specificity of ShaV to sialylated glycans. The selected glycans were determined as decreasing signal intensity according to the virus titer. Relative fluorescence units were calculated using RayBiotech array analysis program. Bars display the average fluorescence units of three measurements. Error bars represent the corresponding SD. (C) List of glycans with decreasing relative fluorescence intensity according to the virus titer. Indicated are the glycan schematic structure and average relative fluorescence units (RFU).

The main purpose of the glycan microarray studies was to investigate the viral binding pattern related to the attachment of sialic acid to the viral HN protein. First, the effective titer of the mouse antiserum against ShaV to be used in the experiment was evaluated. The antibody titer against ShaV was defined as a dilution of antiserum for which a fluorescence signal was obtained by indirect immunofluorescence assay (Fig. S3). Thereafter, glycan microarray was performed using a commercial kit from RayBiotech; the slide contains 4 replicate regions of 300 different sialylated and nonsialylated glycans. Each region was treated with 10^7^ TCID_50_/mL and 10^6^ TCID_50_/mL of ShaV, MARC-145 cell lysate, and sample diluent to enable accurate identification of ShaV binding glycans (Fig. 3A). As shown in Fig. 3B, a pattern of reduced fluorescence signal intensity with virus titer was presented in 14 of the 300 printed glycans. Interestingly, α2,3-linked sialylated glycans showed a higher ShaV binding pattern than other linkages. Among the 8 sialylated glycans with reduced signal intensity according to virus titer, α2,3-linked sialylated glycan was identified in 7 cases, while α2,6-linked sialylated glycan was identified in one case (Fig. 3B and C).

### ShaV binding to α2,3-linked sialylated glycans in the magnetic bead-based virus binding assay.

**FIG 4:**
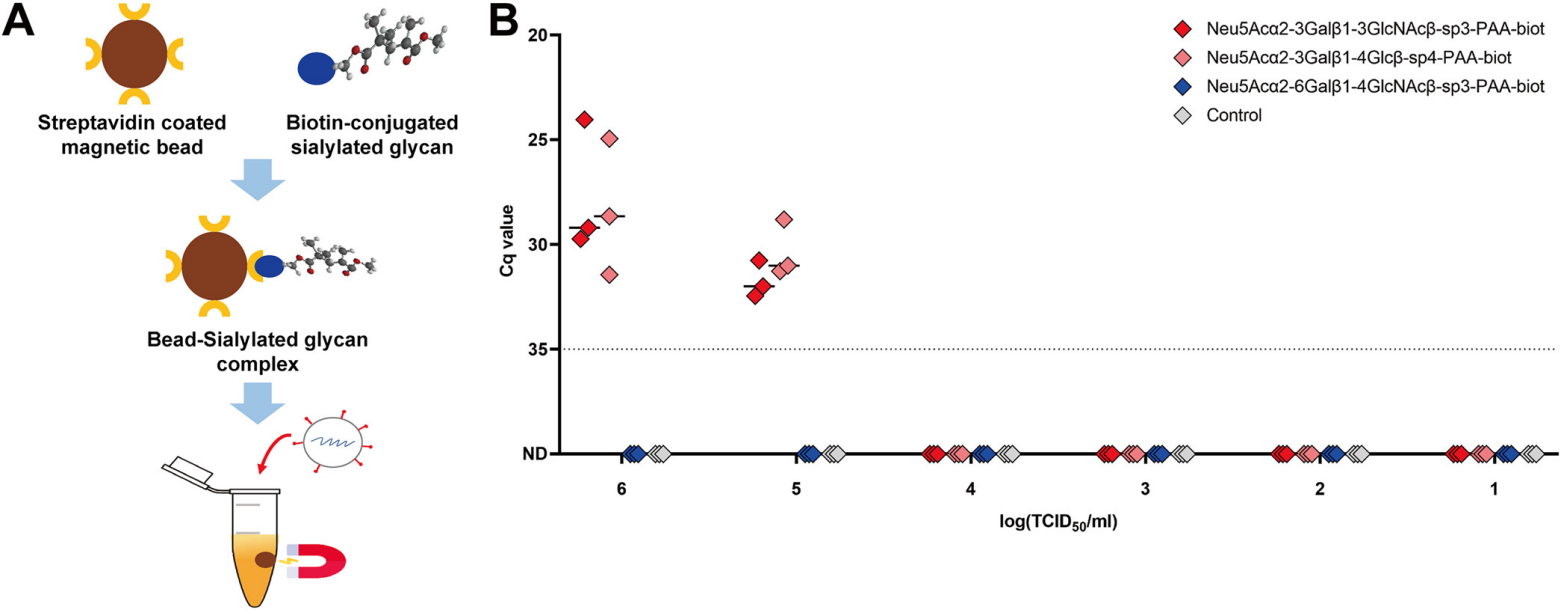
(A) Schematic illustration of magnetic bead-based ShaV binding assay. Streptavidin-coated magnetic beads were incubated with biotin-conjugated sialylated glycans to form bead-sialylated glycan complexes. After incubation, ShaV was added to bead-sialylated glycan complexes. Then, the supernatant and bead complexes were separated using a magnetic separation device. (B) Magnetic bead-based ShaV binding assay efficiency by linked sialic acid types. The bead-sialylated glycan-virus complexes separated using a magnetic separation device were quantified by total viral RNA detection RT-qPCR. Control beads were reacted with glycan resuspension buffer. ND, not detected. The experiments were repeated for three times. Error bars represent the corresponding SD.

The type of sialylated glycans with different sialyl linkages was assessed to determine the interaction of sialic acid with virus in the ShaV binding assay. First, three types of streptavidin T1 bead-sialylated complexes were formed using two α2,3- and one α2,6-linked biotin-conjugated sialylated glycans. Thereafter, ShaV was serial diluted by 10^6^ TCID_50_/mL to 10 TCID_50_/mL and incubated with the bead-sialylated glycan complex (Fig. 4A). At 10^5^ and 10^6^ TCID_50_/mL, ShaV was detected (*C_q_* < 35) in all types of α2,3-linked sialylated glycans. However, real-time reverse transcriptase PCR (RT-qPCR) amplification was not detected for α2,6-linked sialylated glycan as in the control sample (Fig. 4B). In addition, to investigate whether viral passage mutagenesis could affect binding specificity, we compared the results of low (p4) and high passage (p44) numbers. Three mutations (H239Y, A265V, I492T) found at HN protein in low- (p4) and high-passaged (p44) viruses tended to be consistent (Fig. S4A). At 10^5^ and 10^6^ TCID_50_/mL, both low- and high-passaged ShaV were detected in all types of α2,3-linked sialylated glycans except for α2,6-linked sialylated glycans (Fig. S4B).

### Effect of neuraminidase inhibitors on the specificity of ShaV replication inhibition.

**FIG 5:**
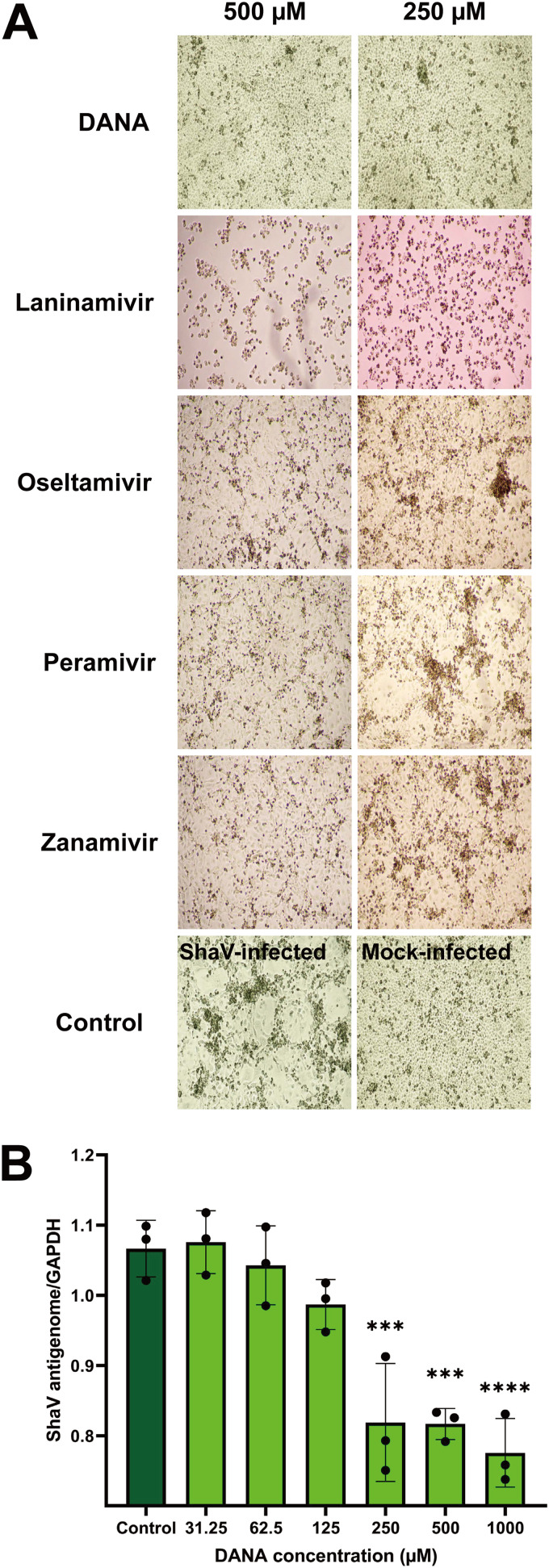
(A) Microscopy observations of ShaV-induced CPE reduction in MARC-145 cells by NAIs. Infected cells were incubated with five different NAIs and were evaluated for CPE at 4 days postinoculation (dpi) by phase-contrast light microscopy (magnification, ×40). (B) Quantitative analysis of ShaV antigenome by DANA. Infected MARC-145 cells reacted with DANA. Control cells were infected with ShaV only. Relative expression of viral antigenome normalized with monkey GAPDH gene quantified by real-time qPCR. The experiments were repeated six times. Error bars represent the corresponding SD. The data were analyzed by one-way ANOVA with Dunnett’s multiple-comparison test. ***, *P* < 0.001; ****, *P* < 0.0001.

To evaluate whether the ShaV HN protein functions not only as a binding protein but also as a neuraminidase, we measured cytopathic effect (CPE) reduction and the levels of viral antigenome present in virus-infected MARC-145 cells treated with five neuraminidase inhibitors (NAIs), namely, laninamivir, oseltamivir, peramivir, zanamivir, and 2,3-dehydro-2-deoxy-*N*-acetylneuraminicacid (DANA). Inhibition of viral replication by NAIs was measured based on a reduction in CPE using light microscopy. Treatment of virus-infected cells with DANA reduced ShaV-specific CPE in a concentration-dependent manner (range, 250 to 1,000 μM). However, laninamivir, oseltamivir, peramivir, and zanamivir did not inhibit CPE at <500 μM (Fig. 5A). The anti-ShaV activity of DANA was assessed based on a reduction of CPE. The CPE was graded as follows: 0, no CPE; 1, 1% to 25%; 2, 26% to 50%; 3, 51% to 75%; and 4, 76% to 100% CPE. The percentage of CPE inhibition was calculated using the equation % inhibition = (CPE of DANA/CPE of virus control) × 100 to determine the 50% effective concentration (EC_50_) of DANA. ShaV-specific CPE was dose-dependently inhibited by DANA, with an EC_50_ of 64.01 ± 10.64 μM (Fig. S5). The inhibition of viral replication by DANA in MARC-145 cells was also evaluated using a CPE reduction assay with crystal violet staining; the presence of a purple color indicated live cells without viral infection, whereas the absence of such color indicated lysis of the host cells by viral infection. A gradient purple color appeared in a concentration-dependent manner (range, 62.5 to 1,000 μM) owing to DANA (Fig. S6). To further determine whether DANA is related to the inhibition of ShaV replication, the *C_q_* value for viral antigenome was quantified and normalized to the *C_q_* value for monkey GAPDH (glyceraldehyde-3-phosphate dehydrogenase). As shown in Fig. 5B, DANA significantly inhibited viral replication in a concentration-dependent manner (range, 250 to 1,000 μM), which is consistent with microscopic observations.

## DISCUSSION

Many paramyxoviruses use sialic acid as a receptor to bind to target cells and initiate infection. For example, members of the subfamilies *Avulavirinae* and *Rubulavirinae* and some *Orthoparamyxovirinae* of the family *Paramyxoviridae* share a feature, as they are viruses that bind to sialic acid-containing cell receptors. These viruses include parainfluenza virus, mumps virus, and NDV, which are human and animal pathogens that bind to host cell-specific receptor via the HN glycoprotein. In general, hemagglutinin is believed to recognize sialic acid, ultimately enabling binding of the virus to the host cell surface. Further, neuraminidase is believed to promote the release of mature virions via receptor removal. Viral binding not only depends on the affinity of HN for terminal sialic acid residues in the host cell but also on the linkage between sialic acid and galactose (22). The binding of sialic acid to the penultimate galactose is considered a determinant of viral binding specificity. Influenza viruses are well-known to have different binding specificities depending on the type of linkage. Avian influenza viruses bind to α2,3-linked sialic acids, while mammalian influenza viruses bind to α2,6-linked sialic acids (23–26). For paramyxovirus, Sendai virus, HPIV1, and HPIV3, belonging to the genus *Respirovirus*, bind to a2,3-linked sialic acids, while HPIV3 binds to α2,3-linked or α2,6-linked sialic acids (15, 27, 28). In addition, NDV belonging to the genus *Orthoavulavirus* can bind to both α2,3-linked and α2,6-linked sialic acids (14). Thus, the specific linkage of sialic acid residues to glycoconjugates is considered to be one of the major determinants of viral tropism.

In this study, we showed, using four types of experiments with different approaches, that ShaV binds sialic acid on cell surface glycoconjugates. First, the interaction of ShaV with sialic acid was demonstrated through the blockade of ShaV infection of MARC-145 cells by sialidase, an enzyme that removes sialic acid residues (29–31). By normalizing the *C_q_* value of ShaV antigenome with the *C_q_* value of monkey GAPDH, the efficiency of virus infection was found to be reduced in α2,3-linked sialic acid-cleaved cells (Fig. 1A). In addition, based on total viral RNA quantification, infection of virus was reduced after sialidase treatment of the α2,3-linked cleavage (Fig. 1B). Plaque reduction assay also showed that cleavage of α2,3-linked sialic acid in MARC-145 cells was involved in regulating the replication of ShaV (Fig. 2A and B). Altogether, the sialidase from Clostridium perfringens, which preferentially cleaves α2,3-linked sialic acid residues, inhibited infection of MARC-145 cells when tested in a dose-dependent manner. These results suggest that ShaV may use α2,3-linked sialic acids as potential receptors.

The binding specificity of ShaV to sialic acids was evaluated using glycan microarray. Glycan microarray analysis is very useful for identifying new receptor motifs; however, it is associated with limitations such as nonspecific binding (32). Therefore, in this study, the distinction between virus binding specific and nonspecific signals was confirmed using viruses with different titers, MARC-145 cell lysates, and sample dilutions. By analyzing the signal reduction based on the virus titer, the virus was confirmed to predominantly bind to α2,3-linked sialic acids (Fig. 3B and C). However, the fluorescence signal value was lower than that reported in other studies, as purification of virus or HN protein was not performed (32–35), and, despite the nonsialylated glycan binding signal, which is thought to be a nonspecific reaction of polyclonal antibodies, our findings suggest the receptor binding pattern of ShaV.

To further confirm the role of α2,3-linked sialic acid binding to ShaV, streptavidin-coated magnetic beads were treated with different biotin-conjugated sialylated glycans. The streptavidin-biotin interaction is stable and highly sensitive. Further, biotin of a small-molecule protein can bind with high affinity to streptavidin of a tetrameric biotin binding protein and rarely interferes with the function of the labeled molecules (36–38). In this study, total viral RNA detection by RT-qPCR was performed using bead-sialylated glycan-virus complexes separated by a magnetic device. Interestingly, only α2,3-linked sialic acids had the ShaV binding signal (Fig. 4B). However, it is possible that viral passage-induced mutations affect binding specificity. Therefore, we performed further magnetic bead-based virus binding assay using low- (p4) and high-passaged (p44) ShaV. Consistent with the results of p17 ShaV, all types of α2,3-linked sialylated glycans were detected in both low- and high-passaged ShaV except for α2,6-linked sialylated glycans (see Fig. S4 in the supplemental material). These results further demonstrate that ShaV binds to α2,3-linked sialic acid rather than α2,6-linked sialic acid and that mutations in low- and high-passaged ShaV do not affect the binding function of HN protein.

HN protein not only binds sialic acids as a receptor but exerts neuraminidase activity to remove sialic acids. In general, the function of neuraminidase in the viral release step is to prevent aggregation of virions by cleaving the bonds between sialic acids on the cell surface and glycoproteins of newly budded virions (22). In this study, ShaV replication was inhibited by the treatment of infected MARC-145 cells with NAIs. Of the five NAIs administered, only DANA-treated cells had reduced viral under light microscopy (Fig. 5A). However, a cytotoxic effect was observed with high concentrations of laninamivir, even in uninfected MARC 145 cells. The structure of DANA was used as a template for the NAIs zanamivir, oseltamivir, laninamivir, and peramivir for the treatment of mutated human influenza virus infection (39). For the paramyxoviruses HPIV and NDV that infect humans, zanamivir displayed significant antiviral activity (40, 41). Although ShaV has the function of neuraminidase, this virus might not be affected by influenza-specific NAIs. Thus, ShaV is speculated to have a different neuraminidase structure.

The EC_50_ value of DANA was 64.01 μM. Further, DANA induced low toxicity in MARC-145 cells (Fig. S5). However, in the CPE reduction assay, DANA inhibited the replication of ShaV in a dose-dependent manner, with a purple color observed when concentrations between 62.5 and 1000 μM were employed (Fig. S6). Such finding does not align with the microscopic observations. Therefore, we assessed the inhibition of ShaV replication at the genetic level. Paramyxoviruses have a negative-sense single-stranded RNA genome. During the replication cycle, the viral genome is transcribed to produce capped and polyadenylated mRNAs. Thereafter, N mRNA is translated, and the accumulation of the N protein leads to full-length antigenome synthesis (42, 43). In this study, antigenome-specific primers were designed for cDNA synthesis and detection via real-time PCR. For antigenome detection only, antigenome-specific primer was designed with a sequence of the intergenic region of nucleocapsid and phosphoprotein to distinguish it from mRNA. By determining the antigenome *C_q_* value of the DANA-treated cells and normalizing this value with the *C_q_* value of monkey GAPDH, virus replication was identified to be inhibited in a concentration-dependent manner (range, 250 to 1,000 μM) as observed under a microscope (Fig. 5B). These results support that ShaV has HN activity, and sialic acid is a potential receptor.

Sialic acid residues are present on the cell surface as sialoglycoconjugates, including glycolipid and glycoprotein. For viruses that bind to sialoglycoconjugates, these structurally distinct types of molecules have been proposed to have different binding specificities (44, 45). Although the specific identity of the receptor molecule in MARC-145 cells is not clear, this study provided evidence of sialic acid binding of a novel paramyxovirus.

Identifying the receptors used by viruses is not only an important task that can aid in the determination of the mechanism of viral infection. In fact, such identification can be very useful for gaining a better understanding of viral tropism. In this study, we demonstrated the interaction of ShaV with sialic acid and revealed that α2,3-linked sialic acids are potential receptors for ShaV infection of MARC-145 cells.

## MATERIALS AND METHODS

### MARC-145 cell culture.

The MARC-145 cell line derived from African green monkey kidney was maintained in Dulbecco’s modified Eagle’s medium (DMEM) supplemented with 5% heat-inactivated fetal bovine serum (FBS) and antibiotic-antimycotic (100 U/mL penicillin, 100 μg/mL streptomycin, and 0.25 μg/mL amphotericin B) at 37°C in a 5% CO_2_ incubator.

### Virus growth and titration.

To amplify ShaV, 3 × 10^6^/mL MARC-145 cells were seeded in a 75-cm^2^ tissue culture flask in DMEM (5% FBS) and incubated overnight to form a monolayer. MARC-145 cells were then infected with ShaV at an MOI of 0.1 and maintained in DMEM (2.5% FBS) for 5 days. The ShaV was serially passaged from low to high passage in MARC-145 cells. ShaV infection was determined by observing CPE under an inverted light microscope. The viral titer was quantified by the Reed-Muench method and expressed as TCID_50_. In this study, all experiments were mainly performed with viruses of the same passage number (p17). We further used low- (p4) and high-passaged (p44) ShaV for magnetic bead-based virus binding assay.

### Sialidase treatment of MARC-145 cells.

MARC-145 cells were treated with sialidase (Roche, Switzerland) from Arthrobacter ureafaciens (10 U/mL), Vibrio cholerae (1 U/mL), and Clostridium perfringens (5 U/mL), following a 2-fold serial dilution from 500 mU/mL in DMEM. Prior to ShaV infection, cell monolayers in 96-well plates were incubated with 100 μL of diluted sialidases at the indicated concentrations for 1 h at 37°C. After washing with Dulbecco’s phosphate-buffered saline (DPBS), cells were inoculated with ShaV at an MOI of 0.1 or 1 for 1 h at 37°C. The mock controls were treated with DMEM only. The cells were then washed with DPBS and maintained in DMEM (2.5% FBS) for 12 h. The experiments were repeated six times (*n* = 6).

### Plaque reduction assay.

The antiviral effect of sialidase treatment was evaluated by a plaque reduction assay. Cells were inoculated with ShaV at an MOI of 0.1 in the same manner as was done for sialidase treatment, and virus was harvested by freeze-thaw after 12 h. Confluent MARC-145 cells seeded into 12-well plates were washed with DPBS, inoculated with the sialidase-treated virus samples, and incubated for 1 h at 37°C. After washing with DPBS, cells were treated with overlay consisting of 0.5% (wt/vol) agarose, 2.5% FBS, 1× antibiotic-antimycotic, and 1× DMEM. The plates were incubated at 37°C in 5% CO_2_ for 7 days, followed by fixation of the cell monolayer with 4% formaldehyde and staining with 0.5% (wt/vol) crystal violet for visualization.

### Glycan microarray.

The binding specificity of ShaV was determined using a glycan array kit (RayBiotech, USA), which contained 300 synthetic glycans, each on four printed glass slides. Sandwich-based detection was performed according to the manufacturer’s protocols. Four printed wells in glass slides were incubated with 400 μL of 10^7^ TCID_50_/mL of the ShaV, 10^6^ TCID_50_/mL of the ShaV, MARC-145 cell lysate, and sample diluent for 3 h at room temperature with gentle rocking. The glass slide was washed with 1× wash buffer I and II. Of note, the sample diluent and wash buffer were provided in the kit. Mouse antiserum against ShaV was used as the primary antibody. The antibody efficiency of antiserum was confirmed via an immunofluorescence assay as previously described (3). The serum was diluted 1:50 with sample diluent. Thereafter, 400 μL was added to each well for 2 h at room temperature except for the well containing sample diluent. After washing with wash buffer, 400 μL of Alexa Fluor 488-conjugated goat anti-mouse IgG secondary antibody (diluted 1:500 with sample diluent) was added to each well for 2 h at room temperature. The glass slides were washed, and the fluorescence signals were detected using a GenePix 4100A microarray scanner (Molecular Devices, USA). Glycan microarray analysis was performed by Gene On Biotech (Daejeon, South Korea).

### Magnetic bead-based ShaV binding assay.

The magnetic bead-based ShaV binding assay comprised the following steps: bead washing, bead-sialylated glycan complex formation, and ShaV binding. Briefly, 10 μL of Dynabeads MyOne streptavidin T1 beads (Invitrogen, USA) was resuspended in a 1.5-mL tube. Thereafter, 200 μL of 1× PBS was added to the tube, which was left to stand for 1 min. Magnetic separation was carried out using MagJet separation rack (Thermo Fisher Scientific, USA) and washed three times with PBS. Neu5Acα2-3Galβ1-3GlcNAcβ-sp3-PAA-biot, Neu5Acα2-3Galβ1-4Glcβ-sp4-PAA-biot, and Neu5Acα2-6Galβ1-4GlcNAcβ-sp3-PAA-biot of biotin-conjugated sialylated glycans (GlycoNZ, New Zealand) were used to form a bead-sialylated glycan complex. The sialylated glycan resuspension buffer was used as a control. After the beads were resuspended in 10 μL PBS, 10 μL (10 ng) of sialylated glycan was added and incubated for 30 min at room temperature. Following incubation, the bead-sialylated glycan complex was washed three times with PBS using a MagJet separation rack. Finally, the virus was 10-fold serially diluted from 10^7^ TCID_50_/mL for ShaV binding with the bead-sialylated complex. The beads were resuspended in 10 μL PBS, and 200 μL of diluted virus was added and incubated for 10 min at room temperature. After incubation, the supernatant and the bead complexes were separated using MagJet separation rack. Total RNA was extracted from the washed beads using TRIzol LS reagent. Viral RNA detection by RT-qPCR was performed using SensiFAST probe NO-ROX one-step kit (Meridian Bioscience, USA).

### MARC-145 cell treatment with NAIs.

MARC-145 cell monolayers in 96-well plates were inoculated with 200 TCID_50_/well of the ShaV and incubated for 1 h at 37°C. Thereafter, the cells were washed with DPBS. To treat MARC-145 cells with the neuraminidase inhibitor, 2,3-dehydro-2-deoxy-*N*-acetylneuraminicacid (DANA) (Sigma-Aldrich, USA), DANA was 2-fold serially diluted from 1 mM in DMEM (2.5% FBS). Other NAIs, namely, laninamivir, oseltamivir, peramivir, and zanamivir, were 2-fold serially diluted from 500 μM in DMEM (2.5% FBS). After washing with DPBS, the diluted NAIs at the indicated concentrations were added to the MARC-145 monolayers, which were then incubated for 4 days at 37°C. The mock controls were treated with DMEM (2.5% FBS) only. The experiments were repeated three times (*n* = 3). Laninamivir, oseltamivir, peramivir, and zanamivir were kindly provided by Min Suk Song (Chungbuk National University College of Medicine, Cheongju, South Korea).

### Quantitative real-time PCR analysis.

For total RNA detection of ShaV attached to MARC-145 cells, the cell samples subjected to sialidase treatment for 12 h were collected via trypsin treatment, and RNA was extracted with TRIzol LS (Invitrogen, USA). ShaV RNA was quantified using the SensiFAST probe NO-ROX one-step kit with specific primers and probe that target regions of the membrane and fusion protein. RT-qPCR was performed using the following cycling conditions: reverse transcription at 45°C (10 min) followed by 95°C (5 min), and then 40 cycles at 95°C (10 s) and 60°C (20 s). For antigenome detection, neuraminidase or sialidase-treated cells were cultured for 4 days and 12 h, respectively. Thereafter, cells were collected via trypsin treatment, and RNA was extracted with TRIzol LS. cDNA was synthesized using a Moloney murine leukemia virus (M-MLV) reverse transcriptase kit (Promega, USA) with a mixture of specific primers, which were designed with the sequence of the intergenic region of the nucleocapsid and phosphoprotein (N-P). ShaV antigenome was detected in the synthesized cDNA using the SensiFAST probe NO-ROX kit with specific primers and probe that target regions of the N-P intergenic region. Real-time PCR was performed using the following cycling conditions: 95°C (5 min) and then 40 cycles at 95°C (20 s) and 60°C (30 s). The antigenome quantification cycle (*C_q_*) value was normalized to that of monkey GAPDH (46). The sequence data of the primers are provided in Table S1 in the supplemental material.
