# Supplementary material for: α2,3-Linked Sialic Acids Are the Potential Attachment Receptor for Shaan Virus Infection in MARC-145 Cells
Source: Microbiol Spectr. 2022 Aug 4;10(4):e01256-22. doi: 10.1128/spectrum.01256-22 (PMC9430483; doi:10.1128/spectrum.01256-22)
Supplement: Supplemental file 1 — Supplemental material. Download spectrum.01256-22-s0001.pdf, PDF file, 0.9 MB [file spectrum.01256-22-s0001.pdf]

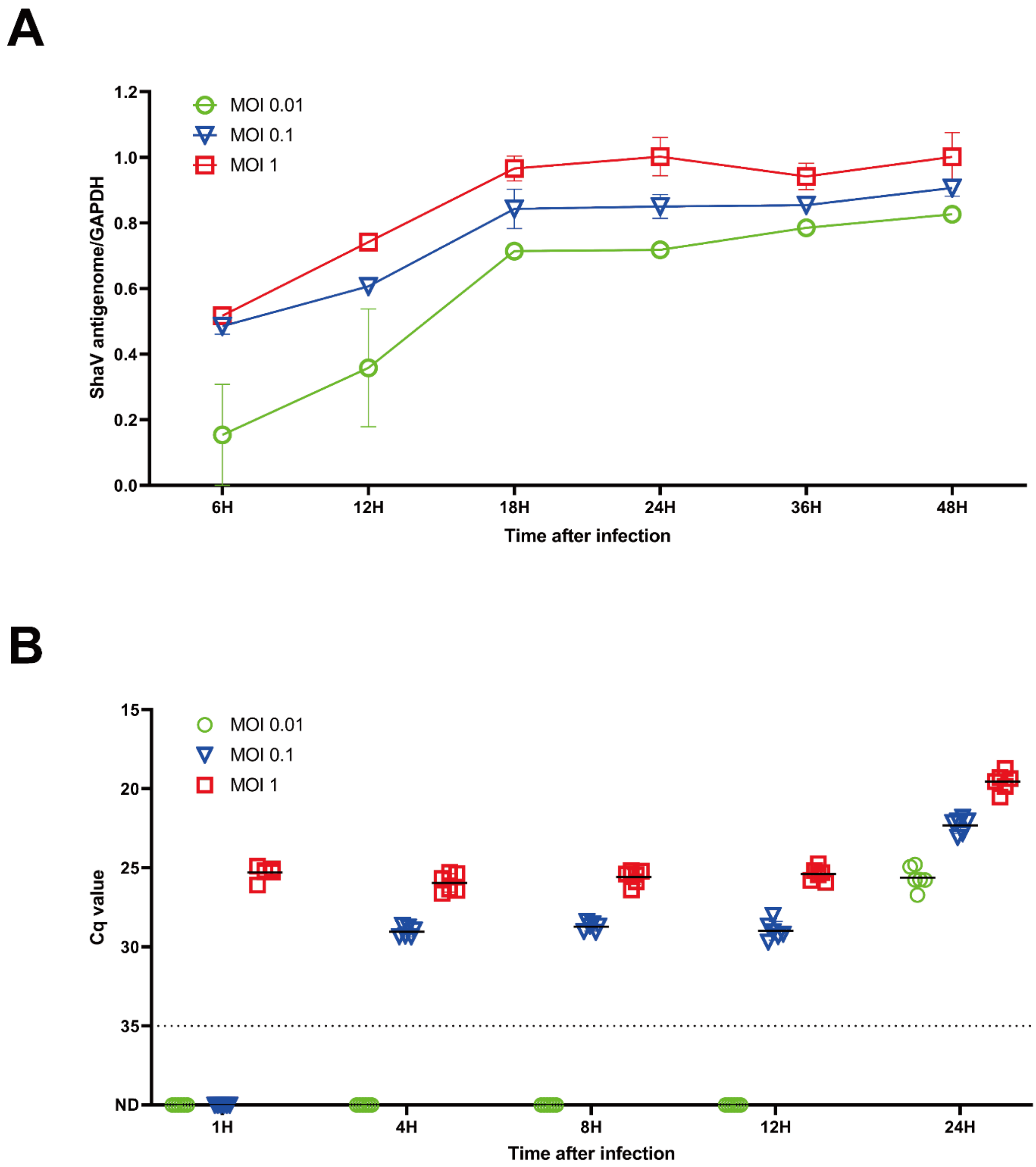

Figure S1. Optimization of time point for antigenome and total ShaV RNAs quantification. MARC-145 cells infected with different virus titers were incubated in a time dependent manner for antigenome and total ShaV RNAs quantification in a sialidase treatment assay. (A) The amount of viral antigenome was plateaued from 18 hpi at MOI of 0.01, 0.1, and 1. (B) The amount of total viral RNAs was increased from 24 hpi at MOI of 0.01, 0.1, and 1. The experiments were repeated for three times. Error bars represent the corresponding SEM.

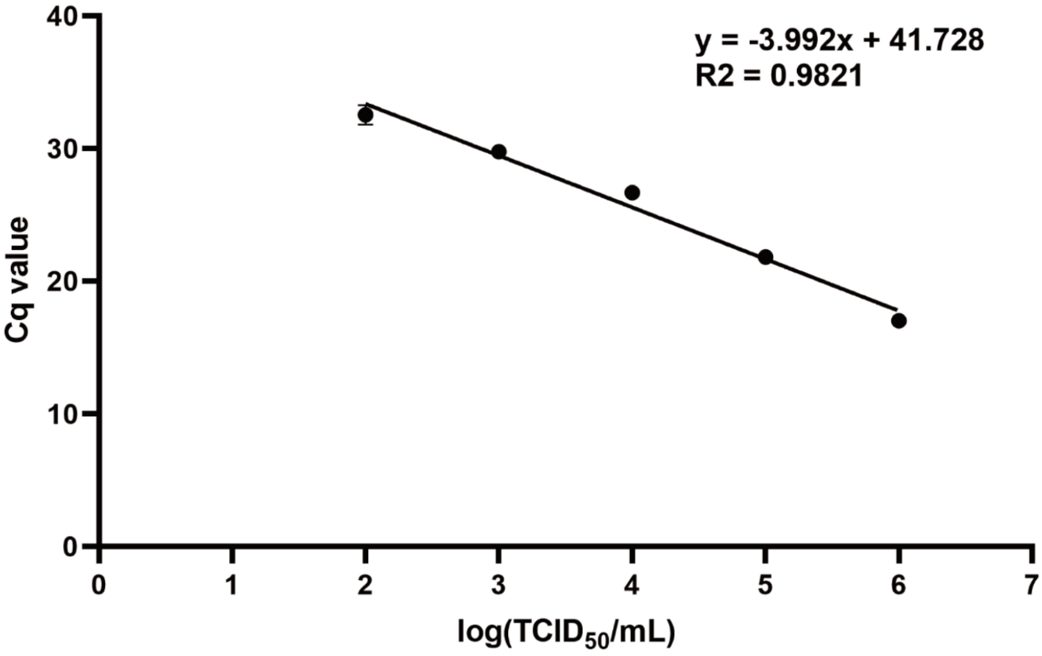

Figure S2. Standard curve of ShaV. Viral RNA was extracted from 10-fold serially diluted virus stock from 10^2 to 10^7 $TCID_{50}$/mL. Then, Cq values were obtained by RT-qPCR in three independent experiments.

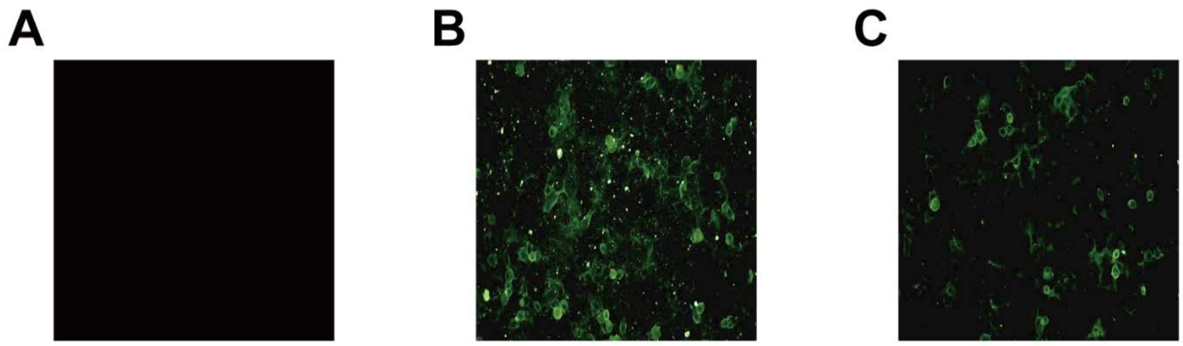

Figure S3. The efficacy of antiserum against ShaV was evaluated by indirect immunofluorescence assay. (A) Mock-infected, (B) ShaV-infected with 10^7 $TCID_{50}$/mL, and (C) ShaV-infected with 10^6 $TCID_{50}$/mL MARC-145 cells reacted with mouse antiserum against ShaV.

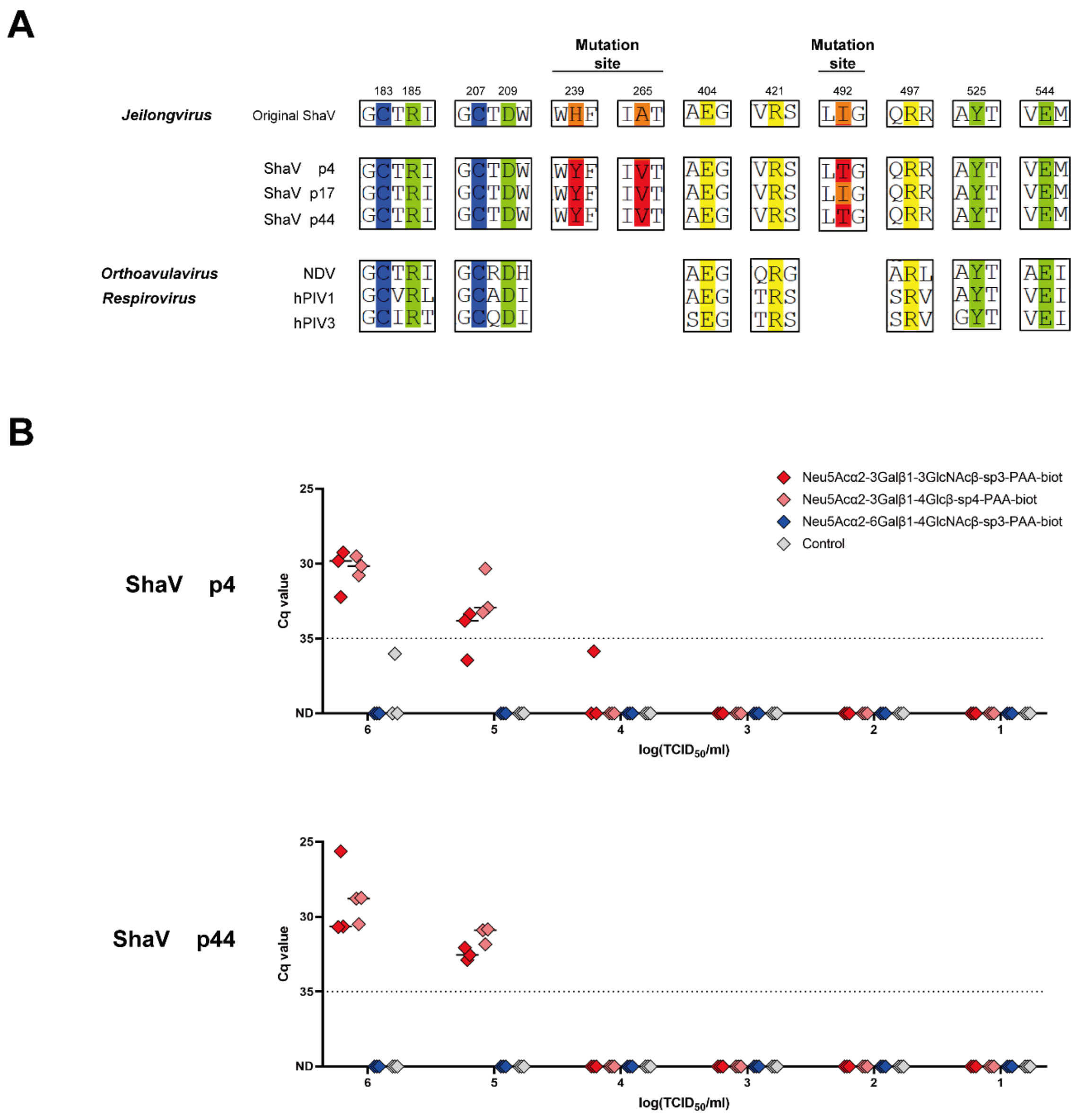

Figure S4. Identification of the correlation between the putative receptor binding site and the HN mutation residues. (A) Three mutations (H239Y, A265V, I492T) found at HN protein in low- (p4) and high-passaged (p44) viruses tended to consistent, whereas two mutations (H239Y, A265V) were identified in p17. The conserved amino acids of putative receptor binding site I (ShaV: R185, D209, E404, R421, R497, Y525, and E544; yellow color) and II (ShaV: C183, R185, C207, D209, Y525, and E544; blue color) were identified through sequence alignment using BioEdit software. Green residues indicate commonly conserved amino acids in receptor binding site I and II of ShaV, NDV, and hPIV. (B) All types of α2,3-linked sialylated glycans were detected in both low- and high-passaged ShaV except for α2,6-linked sialylated glycans. ND, not detected. The experiments were repeated for three times. Error bars represent the corresponding SD.

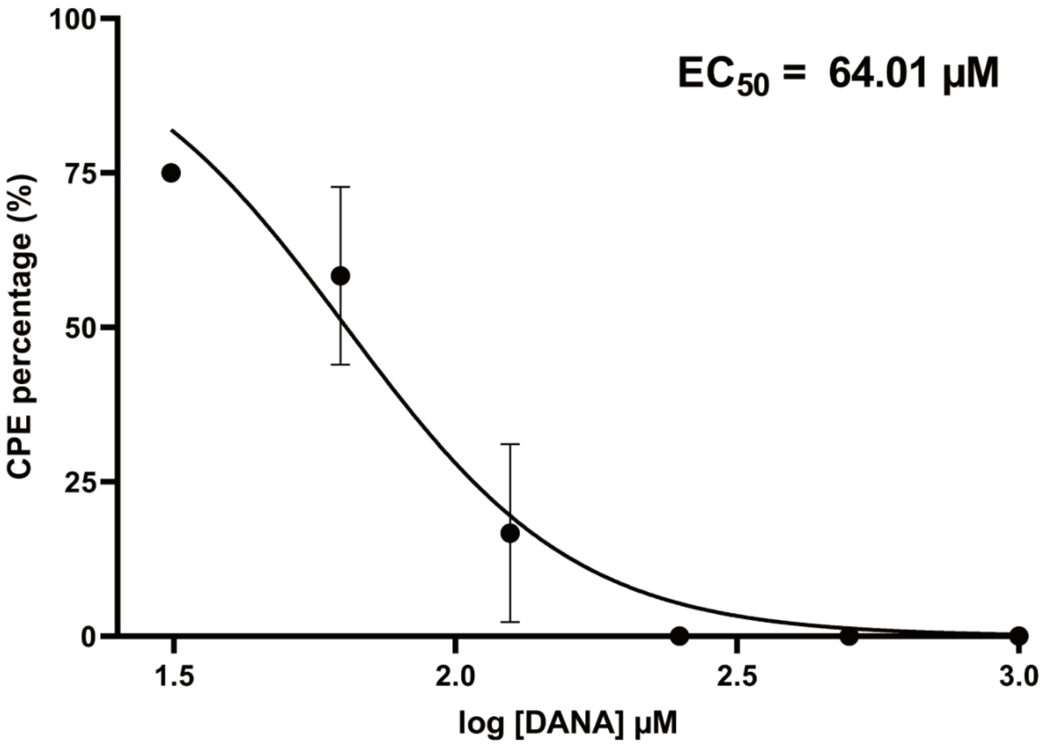

Figure S5. Evaluation of EC_{50} values through inhibition of ShaV replication by DANA. The percentage level of CPE was determined through microscopic observation according to the DANA concentration. EC_{50} was calculated using GraphPad Prism 9.2.0. The experiments were repeated for three times. Error bars represent the corresponding SD.

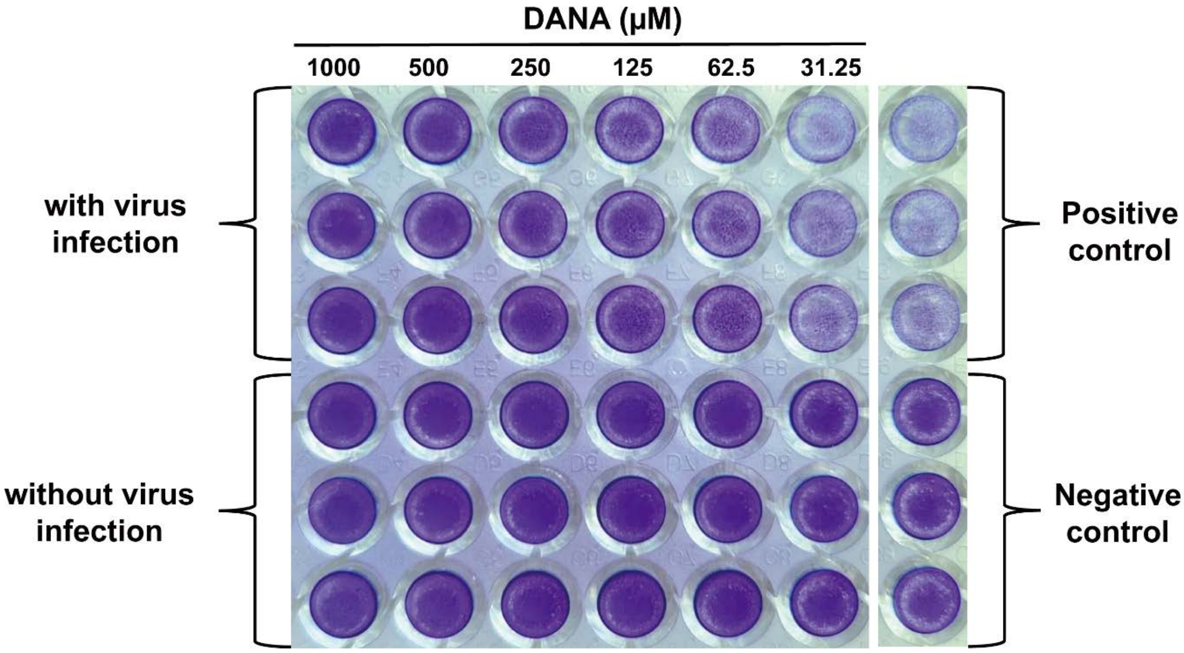

Figure S6. CPE reduction assay of ShaV at different DANA concentrations. ShaV-infected MARC-145 cells reacted with different DANA concentrations. Positive and negative control refer to ShaV-infected and mock-infected MARC-145 cells, respectively. The cells are stained with crystal violet after incubation. Absence of purple color indicates that the infecting virus causes lysis of MARC-145 cells.

Table S1. Primer information of qPCR.

Target	Primer	Sequence (5' to 3')	PCR condition	Reference
ShaV antigenome	cDNA syn	GAGCATCCAGAGACTTCCAGTGACGTTTGGCTTTGTCTG	70 min at 42 ℃<br>10 min at 70 ℃	—
	NP-F	GGATTAACCTCAAAACCAATGC	5 min at 95 ℃<br>40 cycles: 20 s at 95 ℃, 30 s at 60 ℃	
	NP-R-tail	GAGCATCCAGAGACTTCCA		
	NP-probe	FAM-AAAGTGACATAGGCCAAGCTC-BHQ1		
ShaV whole RNAs	PVM-F	CCCAGGAGTATGGTTATCAAGTGAGG	10 min at 45 ℃<br>5 min at 95 ℃<br>40 cycles: 10 s at 95 ℃, 20 s at 60 ℃	[1]
	PVM-R	TCCATTGGGCTCTCTTTGTTTGC		
	PVM-probe	FAM-CCCATCCCAGACCAGCCACCAGACCC-TAMRA		
Monkey GAPDH	GAPDH-F	TGACAACAGCCTCAAGATCG	5 min at 95 ℃<br>40 cycles: 20 s at 95 ℃, 30 s at 60 ℃	[2]
	GAPDH-R	GTCTTCTGGGTGGCAGTGAT		
	GAPDH-probe	HEX-TGGAAGGACTCATGACCACA-3IABkFQ		

References

1. Noh, J. Y. et al. Isolation and characterization of novel bat paramyxovirus B16-40 potentially belonging to the proposed genus Shaanvirus. Sci Rep 8, 12533, doi:10.1038/s41598-018-30319-7 (2018).
2. Shi, X. et al. Nonstructural protein 11 (nsp11) of porcine reproductive and respiratory syndrome virus (PRRSV) promotes PRRSV infection in MARC-145 cells. BMC Vet Res 12, 90, doi:10.1186/s12917-016-0717-5 (2016).
